# Phagocytosis: a repertoire of receptors and Ca^2+^ as a key second messenger

**Published:** 2014-08-01

**Authors:** Alirio J. Melendez, Hwee Kee Tay

Volume 28, issue 5 (2008), pp. 287–298

Following an investigation by the National University of Singapore into the publications of A.J. Melendez, it has been brought to the attention of the Editorial Board that a portion of the text in the above Review Article (the first six sentences of the Introduction) was used without appropriate reference to the original source from Dr Melendez's previous publication in 2005 entitled ‘Calcium Signaling during Phagocytosis’ appearing in *Molecular Mechanisms of Phagocytosis* [Chapter 9, pp. 117–132. (Rosales, C., ed.), Springer, Science].

